# Health numeracy skills of medical students:cross-sectional and controlled before-and-after study

**DOI:** 10.1186/s12909-019-1902-6

**Published:** 2019-12-21

**Authors:** Ivan Buljan, Ružica Tokalić, Matko Marušić, Ana Marušić

**Affiliations:** Department of Research in Biomedicine in Health, University of Split School of Medicine, Šoltanska 2, 21000 Split, Croatia

**Keywords:** Health literacy, Health numeracy, Medical education, Medical research

## Abstract

**Background:**

Although numeracy, defined as understanding and handling numbers, is an important skill for the medical profession, it is not clear whether it changes during graduate medical education and whether it can be improved by specific interventions. The objective of this study was to assess objective and subjective numeracy levels at different stages of medical education and explore whether a research methodology/statistics course improves numeracy levels in a longer period.

**Methods:**

We performed cross-sectional and controlled before-and-after studies. First-year sociology students and first- to sixth-year medical students from the in the cross sectional study and two groups of first-year medical students in a controlled before-and-after study. The intervention was a course on biostatistics and research methodology using blended approach. Numeracy was measured using Subjective Numeracy Scale (Cronbach α = 0.70) and Numeracy Understanding in Medicine instrument (Cronbach α = 0.75).

**Results:**

Whereas first-year medical students did not differ from first-year sociology students in objective numeracy, medicine students had higher results on subjective numeracy. Students from higher years of medical school had generally higher subjective and objective numeracy scores. In the controlled before-and-after study, the intervention group improved more in subjective numeracy (median difference on a 0–8 scale = 0.5, 95% CI 0.3 to 0.7 vs − 0.4, 95% CI − 0.4 to − 0.1, *P* < 0.001) but not in objective numeracy.

**Conclusions:**

Although the numeracy levels at the beginning of the medical school are within the range of non-medical population, both objective and subjective numeracy improve during the higher years of medical school. Curriculum during medical school may help in numeracy increase, while research methodology training may help to increase subjective but not objective numeracy skills.

## Background

Although health literacy is an important predictor of health status [1], it seems that a small percentage of people are adequately health-literate [2]. Taking into account that it is difficult for the general population to follow the development of treatments in medicine, the very idea of health literacy is very broad and includes different concepts [3]. These concepts range from understanding health information and clear comprehension of health risks to performance of basic mathematical operations in the health context, making the entire process of information uptake and translation into behavior very complexed and hard to follow. Recently, health numeracy has received attention as an application of numerical information in the health context. Two reviews of literature demonstrated that the field of health numeracy was not well explored with only few interventions for improving health numeracy levels were developed, usually without validated measures and rarely targeting health professionals [4, 5].

Repeated testing has recently been demonstrated as an effective method to increase the understanding of risk among students [6], while numeracy levels tend to increase after courses using blended learning approach [7–9]. However, to the best of our knowledge, the effects of the numeracy intervention were not explored after longer periods of time using a standardized numeracy test.

The aim of our study was to compare objective and subjective numeracy levels between non-medical population and medical students in different levels of medical education using a cross sectional approach and to explore whether a blended course on research methodology could improve subjective and objective numeracy among undergraduate medical students after 3 months’ period using a controlled before-and-after approach.

## Methods

### Study design

We used a cross sectional approach to study numeracy skills in non-medical student population (sociology students) and in medical students enrolled in the 1st, 3rd^,^ 4th, 5th and 6th year of a graduate medical curriculum. We also used a controlled before-and-after design to examine the effectiveness of a research methodology course in increasing numeracy skills among first-year medical students. The participation in the study was voluntary and participants were not rewarded for the participation. Each participant had to be enrolled in the current academic year for the first time, because including students who repeated a part or the whole study year could affect the validity of the results because they had probably been exposed to more numeracy-related content compared to those who were enrolled for the first time. Each participant used a personalized code for pseudo anonymization of the responses. The code consisted of the elements that could be easily remembered for the next measurement: the first letter of their mother’s name, the first letter of their father’s name, the first letter of the participants’ name, the last two digits of their year of birth and the first letter of their place of birth. In this way, it was possible to pair the participants’ scores in repeated measurements.

### Setting

#### Cross sectional study

Medical students of the 1st, 3rd, 4th, 5th and 6th study year were tested in October 2017 at the time of enrollment in the 2017/2018 academic year. Second-year medical students were not included in the cross sectional study because they were tested twice with the same instruments in the controlled before-and-after study, and therefore their results could not be comparable to other years, who were tested only once. We also tested a group of sociology students from the University of Split Faculty of Humanities and Social Sciences before their first lecture in statistics, at the beginning of their first year. This group served as a non-medical control in order to compare the baseline results of medical students with a population of students who were not enrolled in a medical program but had similar high school education and national high school qualification exams [10]. This baseline control group served to establish whether medical students already come to medical school with higher numeracy level compared to a student group with different education profile.

First-year students had finished high school 3 months before the beginning of the medical school. In order to enroll to the medical school or the faculty of social sciences, they had to complete high school and the pass a national standardized test in order to qualify for university enrolment. High school education does not have formalized education in statistics and probability; these themes are covered by general mathematics education.

#### Controlled before-and-after study

In June 2017, the first-year medical students (2016/2017 enrolment generation) at the University of Split School of Medicine (USSM) were tested at the first day of their research methodology course, at the end of the 2016/2017 academic year. The participants were tested again 2 weeks later, on the last day of the course, and 3 months after, after the summer break – at the beginning of the 2017/2018 academic year. Similarly, the first-year students of the 2017/2018 academic year (non-intervention group) were tested after the introductory lecture on the first day of their first year, then 2 weeks later during the Biochemistry course, and again after 3 months at the beginning of their Anatomy course. The results from the first testing of first-year medical students were also used in the cross-sectional study, as they were tested in the same conditions as the third- to sixth-year students.

### Description of the intervention

At the USSM, research methodology is a part of a vertically integrated evidence-based medicine (EBM) course, consisting of three separate courses held during the first 3 years of a six-year medical graduate program [11]. Graduate medical curriculum lasts for 6 years and enrolls students after high-school education.

The intervention in the controlled before-and-after study was the first-year course (2 weeks’ duration) comprised of 50 class hours of blended learning approach with combination of lectures, seminars and practical exercises in biostatistics and research methodology using face-to-face approach and online Modular Object Oriented Dynamic Learning Environment (Moodle) [9, 11]. The expected competencies gained after this first-year course are basic understanding of research methodology in medicine, critical evaluation of scientific reports, and understanding and application of basic biostatistics [11].

### Variables

We collected the data about the participants’ sex, age and high-school grade point average (GPA). Previous research indicated that numeracy levels can be measured as subjective and objective construct, and that there is no evidence for optimality of one approach over another [12]. In order to grasp a wide range of numeracy skills and perceptions, we used two numeracy assessment tools: 1) subjective – where numeracy is self-assessment of numeracy based on the perception of one’s abilities and/or preferences of numerical information, and 2) objective – assessing the ability to perform numerical tasks and interpret numerical information. The set time to complete both measures was 25 min at all measurement times and for all groups.

1. *Subjective Numeracy Scale* [13] is a self-report measure where one of the subscales measures perceived ability to perform various mathematical tasks and the second one measures the preference for the use of numerical information over prose information. It consists of 8 questions, and the task of the participant is to assess his or her ability/preference considering numerical information on a scale from 1 to 6, where the higher result indicates higher subjective numeracy levels. The final score is the average of the answers (theoretical range 1–8) [14]. The reliability of the scale in our study was α = 0.70 (95% confidence interval (CI) = 0.65 to 0.75).
2. *The Numeracy Understanding in Medicine instrument* – NUMi [15] is a measure of objective numeracy, which consists of 20 numerical tasks placed in health context, with multiple choice answers. The task for the participant is to choose the correct result out of four possibilities offered. The instrument measures health numeracy in four different domains: *Number sense*, *Probability*, *Statistics* and *Tables and Graphs.* The final score is the sum of correct answers (theoretical range 0–20). The reliability of the scale in our study was α = 0.75 (95% CI = 0.70 to 0.79).

Both tests were translated by one of the authors (IB) from English to Croatian and back-translated by a professional translator. No inconsistencies between the translations were found. Names of the characters in the scenarios described in numeracy tests were replaced with Croatian names, in order to improve content validity.

### Study size

We calculated the study sample size based on the data from previous research [16] which compared participants’ numeracy levels using the 6 item General Health Numeracy Test (GHNT) [17], where participants from Faculty of social sciences had Mean (M) of 1.8 and Standard deviation (SD) was 1.20, and medical students had M = 3.4 (SD = 1.43). Using the online sample size calculator (https://www.stat.ubc.ca/~rollin/stats/ssize/n2.html) with α level set at 0.05 and power at 0.80, we estimated at least 13 participants per group.

### Statistical analysis

We used Kolmogorov-Smirnov test to test the numerical variables for the normality of distribution. Due to the non-normality of the distributions, the data were presented as medians with 95% confidence intervals. Sex differences between the groups were tested using chi-squared test. The differences between groups on age, grade point average (GPA), and the results of subjective and objective numeracy measures were tested with Kruskal-Wallis test and Conover post hoc test if there were more than two independent groups. Mann-Whitney test was used to compare the differences between intervention and non-intervention groups. Friedman test was used for repeated measurements, with Conover post hoc comparison. In a complementary analysis, we used Bayesian ANOVA if there were more than two independent groups and Bayesian Repeated Measures ANOVA for repeated measurements. All statistical procedures were calculated using JASP 0.8.3.1 (JASP Team, 2017) and Bayes Factors were calculated assuming a default prior distribution [18]. Bayes Factors (BF_10_) with values above three even after sequential analysis and robustness check indicated substantial evidence for the alternative hypothesis [19]. In cases of discrepancies between frequentist and Bayesian statistics, we used the Bayesian approach to interpret the significance of the results.

## Results

### Cross-sectional study

In total, 272 students participated in cross sectional study, out of 388 eligible students (response rate = 70.1%). There was no difference between study years in the response rate (Table 1). The only difference in baseline characteristics was an expected older age for higher study year students (Table 1).

**Table 1:** Demographic characteristics of Sociology students from the Faculty of Social Sciences and students of the first, third, fourth, fifth and sixth year of medical studies (*N* = 272)

	Sociology (*n* = 27)	Medicine 1st year (*n* = 54)	Medicine 3rd year (*n* = 54)	Medicine 4th year (*n* = 52)	Medicine 5th year (*n* = 39)	Medicine 6th year (*n* = 46)	P*
Percentage of participants from eligible populations	100%	60%	75.0%	72.2%	64.0%	68.7%	0.541
Female sex (%)	24 (88.9%)	40 (67.7%)	40 (74.1%)	38 (73.1%)	27 (71.1%)	30 (65.2%)	0.306
Age in years (median, 95% confidence interval)	19.0 (18.0 to 19.5)	19.0 (18.0 to 19.0)	21.0 (20.0 to 21.0)†	22.0 (21.0 to 22.0)†	23.0 (23.0 to 23.0)†	23.0 (23.0 to 24.0)†	< 0.001
Grade point average at the end of high school (median, 95% confidence interval)	4.1 (4.0 to 4.3)‡	4.8 (4.7 to 4.8)	4.8 (4.7 to 4.9)	4.7 (4.6 to 4.8)	4.8 (4.7 to 4.9)	4.8 (4.5 to 4.9)	0.109

*Chi-square test for categorical variables and Kruskal Wallis test for continuous variables

†Significantly different from 3rd, 4th, 5th and 6th year groups, Conover post-hoc test

‡Significantly different from other groups, Conover post-hoc test

In Croatian higher education system, grade point average ranges from 2.0 (sufficient) to 5.0 (outstanding)

Sociology students had significantly lower subjective numeracy scores compared to other five groups, and first-year medical students had the lowest subjective numeracy score of all medical school groups (Table 2). On the other hand, there was no difference between the first-year medical students and sociology students on objective numeracy results (Table 2). Finally, fourth, fifth- and sixth-year medical students were always superior to first- and third-year students (Table 2).

**Table 2:** Subjective and objective numeracy scores (median, 95% confidence interval) among students from the Faculty of Social Sciences and students of the first, third, fourth, fifth and sixth year of medical studies (*N* = 272)

Numeracy test	Sociology (*n* = 27)	Medicine 1st year (*n* = 59)	Medicine 3rd year (*n* = 54)	Medicine 4th year (*n* = 52)	Medicine 5th year (*n* = 39)	Medicine 6th year (*n* = 46)	P†	BF_10_‡
Subjective^a^	3.5 (3.1 to 3.6) §	4.8 (4.6 to 5.0)	4.6 (4.4 to 4.9) ‖	4.9 (4.7 to 5.2)	5.0 (4.9 to 5.1)	5.0 (4.9 to 5.1)	< 0.001	2.758 × 10^15^
Objective^a^	15.0 (15.0 to 16.0) #	17.0 (15.0 to 17.0)**	17.0 (16.0 to 17.0) ‖	18.5 (17.0 to 19.0)	19.0 (18.0 to 20.0)	19.0 (18.0 to 19.0)	< 0.001	2.359 × 10^10^

^a^Subjective numeracy score is expressed as mean of answers to eight Likert type questions ranging 1–6; Objective numeracy is expressed as sum of correct answers on a scale from 0 to 20

†Kruskal Wallis test

‡Bayesian one-way ANOVA; BF – Bayes Factor

§Statistically significant from others, post-hoc Conover Iman test

¶Statistically different from Sociology students only

‖Statistically different from Sociology and Medicine 4th, 5th and 6th year groups

#Statistically different from Medicine 3rd, 4th, 5th, 6th year groups

**Statistically different from Medicine 4th, 5th, 6th year groups

### Controlled before-and-after study

**Fig. 1:**
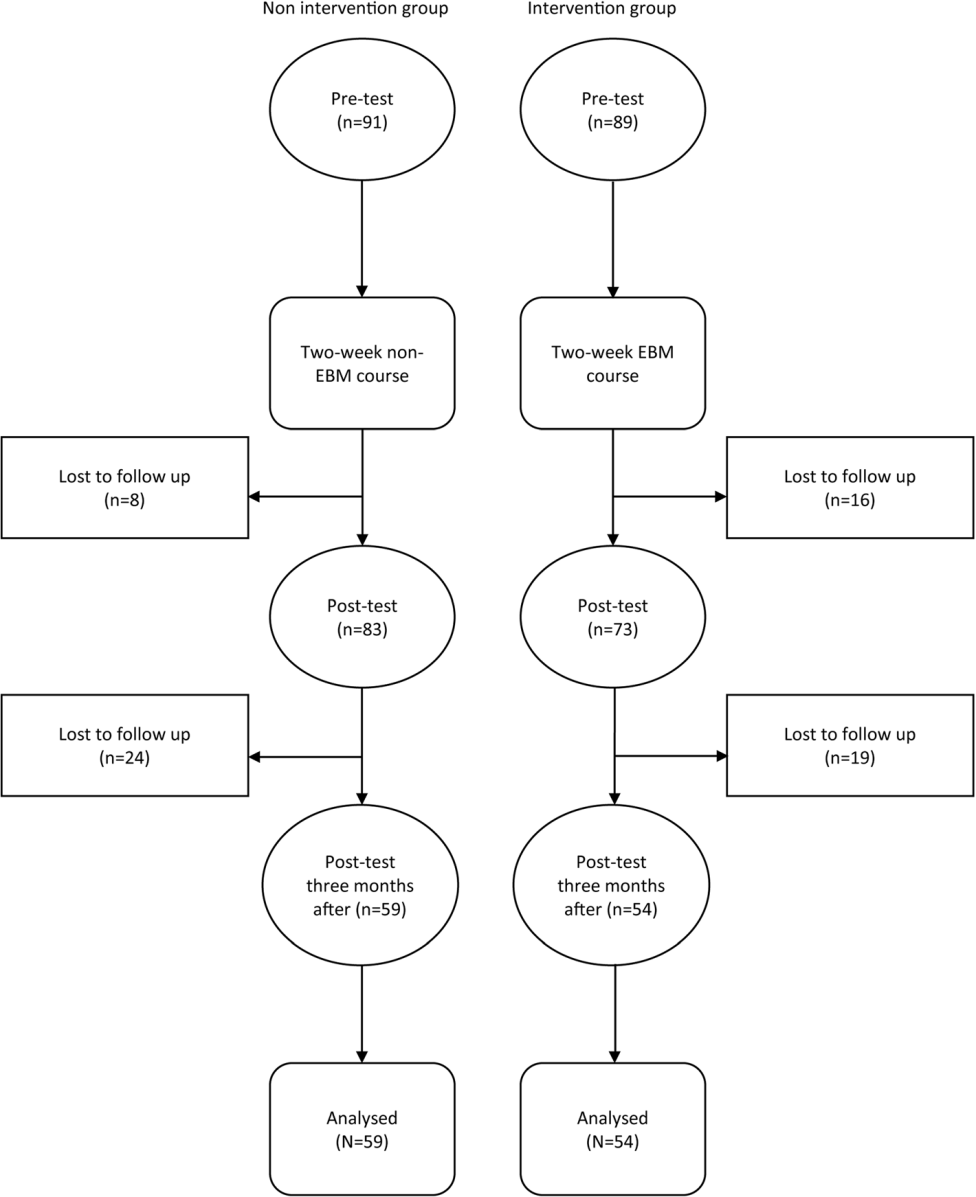
Flowchart of participants who were part of controlled before-and-after study

In total, 113 participants completed all three measurements (54 in the intervention and 59 in the non-intervention group) (Fig. 1). There were no differences between the intervention and non-intervention in sex distribution and grade point average (Table 1). The non-intervention group was significantly younger than the intervention group at the time of measurement, because they were tested at the beginning of the academic year (October), whereas the intervention group was tested at the end of their first academic year (June) (Table 3).

**Table 3:** Demographic characteristics non-intervention (*n* = 59) and intervention group (*n* = 54) of medical students participating in the study

	Non-intervention group (*n* = 59)	Intervention group (*n* = 54)	P*
Females (%)	40 (67.7%)	36 (66.7%)	0.949
Age in years (median, 95% confidence interval)	19.0 (18.0 to 19.0)‡	19 (19.0 to 19.0)	< 0.001
Grade point average at the end of high school (median, 95% confidence interval)^b^	4.8 (4.7 to 4.8)	4.9 (4.8 to 4.9)	0.377

*Chi square for categorical variables and Mann Whitney test for continuous variables

‡In Croatian higher education system, grade point average ranges from 2.0 (sufficient) to 5.0 (outstanding)

Subjective numeracy was higher in the non-intervention group compared to intervention group at the first measurement, but it significantly decreased over time, resulting in intervention group scoring higher on the third measurement (Table 4). Moreover, non-intervention group reached the same level of numeracy scores on their third measurement as the baseline results of the intervention group both on subjective (*P* = 0.301) and objective numeracy (*P* = 0.191). However, comparison of the differences between the third and the first measurement revealed that subjective numeracy decreased in the non-intervention group (mean difference (Md_diff_) = − 0.4, 95% CI = -0.4 to − 0.1), and significantly increased in the intervention group (Md_diff_ = 0.5, 95% CI = 0.3 to 0.7). On the other hand, the non-intervention group showed greater increase in objective numeracy results (Md_diff_ = 2.0, 95% CI = 1.0 to 3.0) compared to the intervention group (Md_diff_ = 1.0, 95% CI = 0.0 to 1.0**)**.

**Table 4:** Objective and subjective numeracy scores (median, 95% confidence interval) in non-intervention medical (*n* = 59) and intervention medical (*n* = 54) student groups

Numeracy test	Group	First measurement	Second measurement	Third measurement	P†	BF_10_‡
Subjective numeracy^a^	Non-intervention group	4.8 (4.6 to 5.0) §	4.5 (4.3 to 4.8)	4.4 (4.3 to 4.8)	< 0.001	990.99
	P¶	< 0.001	0.142	0.029		
	Intervention group	4.5 (3.8 to 4.8)	4.3 (4.0 to 4.5)	4.9 (4.5 to 5.1)	0.168	0.87
Objective numeracy^a^	Non-intervention group	17.0 (15.0 to 17.1) ‖	18.5 (18.0 to 19.0)	19.0 (18.0 to 19.0)	< 0.001	1.49 × 10^10^
	P¶	< 0.001	0.002	0.117		
	Intervention group	18.0 (18.0 to 20.0) #	19.0 (19.0 to 20.0)	19.0 (19.0 to 20.0)	< 0.001	1212.78

^a^Subjective numeracy score is expressed as mean of answers to eight Likert type questions ranging 1–6; Objective numeracy is expressed as sum of correct answers on a scale from 0 to 20

†Friedman non-parametric test for repeated samples

‡Bayesian repeated measures ANOVA; BF – Bayes Factor

§Significantly different from the third measurement, Conover post-hoc test

¶Mann Whitney test for independent samples

‖Significantly different from other two time point measurements, Conover post-hoc test

#No differences were found between different time point measurements on post-hoc testing

## Discussion

The cross sectional analysis in our study demonstrated that there are differences in numeracy levels between different years of medical curriculum. Higher years generally had higher both subjective and objective numeracy then lower years. Also, medical students do not seem to come to the medical school with already high numeracy skills, as they did not differ from first-year sociology students in objective numeracy. However, they have more confidence in their numerical skills, and their subjective numeracy scores were significantly higher than their first-year counterpart at the Faculty of Humanities and Social Sciences. A course in research methodology and statistics further increases their confidence in their numeracy skills, as in the controlled before-and-after study the intervention group of first-year students did not differ in objective numeracy scores compared to the non-intervention group which did not attend the course, but their subjective numeracy scores were significantly higher even 3 months after the course.

The results of our study have to be interpreted in view of several limitations. Due to the fact that the test used in this study measures basic health numeracy, the distribution of the results was skewed, which resulted in a ceiling effect for the participants with higher numeracy levels. We addressed this possible bias by determining changes in numeracy scores for each individual participant and comparing these differences between the groups. Moreover, the differences between the groups were small, often with only few points of difference between the groups, posing a question of their practical relevance. Finally, there could be differences between the groups in terms of motivation and/or readiness to learn numeracy skills at the beginning of the study because students were not aware of their deficit in objective numeracy levels, which could affect later results. Future research should consider assessing this construct at the pre-test stage, using focus groups, interviews or testing, to make the participants aware of their numeracy skills before the intervention, which could make them more motivated for learning.

To the best of our knowledge, this is the first study that assessed the development of health numeracy during medical education using both subjective and objective numeracy assessment and by testing for both short- and long-term retention of numeracy skills. Also, this is the first study that compared numeracy levels of future physicians with a population that had a different educational profile (in this case, sociology students). We tested sociology students only at the baseline because further testing of numeracy levels among sociology students would add complexity to the experimental model because sociology students have different course schedule and attend statistics course throughout the semester (sociology students have 90 h of statistics throughout the year, compared to a 50-h, two-week modular course in biostatistics and research methodology for first-year medical students), and different baseline attitudes towards numerical concepts. Thus, comparison of medical and sociology students would not be possible after 3 months because different factor could determine their numeracy competencies.

First-year medical students had similar results on objective numeracy compared to sociology students, indicating that students of different disciplines at the beginning of their studies have the same levels of objective health numeracy. This is probably the result of the similarity of their high-school education, as most university students come from grammar schools and have to pass the same national examination as a qualifying exam for university entrance [10]. On the other hand, sociology students had lower levels of subjective numeracy compared to medical students’ groups, which may be expected from students who are more oriented towards humanities and social sciences than from medical students who are oriented towards natural sciences. We could not identify studies comparing subjective and objective numeracy levels between different academic disciplines, so future research should explore this knowledge gap.

Our study extends previous research that assessed objective and subjective numeracy in a cross-sectional study design and showed that there were no differences in numeracy skills assessed using either objective or subjective approach [12, 20, 21]. Our study tested an intervention to improve subjective numeracy using a controlled before and after study design and tested both a short-term effect (immediately after the intervention) and a long-term effect (3 months after the intervention).

Furthermore, our study is, to the best of our knowledge, the first use of NUMi for the population of medical students. NUMi is a very broad and sensitive measure of health numeracy (15), which could be the reason why we captured small but significant differences between the study groups. Considering that NUMi has been developed for the general population, the scores in our study were high in comparison to the lay population [15]. We are not aware of a validated and standardized test that would capture higher-level objective numeracy such as may be expected from health professionals in evidence-based practice.

In the controlled before-and-after study, the non-intervention group had significantly improved scores on objective numeracy test compared to its basic scores 3 months before the intervention. It could be argued that this difference was a consequence of frequent exposure to numeracy testing because the scores improved in both non-intervention and intervention group after the initial measurement. Although the non-intervention group had lower results compared to the intervention group at initial measurement, the scores of non-intervention group at the third measurement were not different from the baseline result of the intervention group. Taking into account that the non-intervention group at their third measurement was at the same curricular stage as the intervention group at the baseline, it is possible that the absence of difference indicates that the improvement of health numeracy was due to the first-year curriculum. First-year medical students at the Medical School in Split attend courses in biophysics and biochemistry in their first months of training, where they have to perform numerical calculations embedded in an abstract rather than concrete medical content. It is possible that their basic objective numeracy is improved because of numerical exercises they performed for these courses [6]. However, considering the fact that numbers they worked with were embedded in a non-medical context and represented abstract definitions, subjective numeracy decreased and remained low during the Anatomy and Histology and Embryology courses, which do not contain numerical applications. Further improvement in objective numeracy was observed in the intervention group after a mandatory research methodology and statistics course. It is possible that embedding numerical expressions in medical context resulted in the finding that subjective numeracy levels remained unchanged after 3 months post-course period, while at the same time subjective numeracy decreased in the non-intervention group, which did not have a course where numerical expressions were embedded in a medical context. However, research methodology and statistics course made very little practical difference in students’ objective numeracy levels; which is often the case with individuals who have already high numeracy levels at the baseline [6]. However, it is possible that more frequent courses in biostatistics and research methodology with numerical concepts in a medical context throughout the entire medical curricula could improve students’ attitudes towards use and understanding of numerical concepts in everyday medical work.

The differences between the third and higher years of medical school in the cross sectional study should be interpreted in view of medical curriculum at the Medical School in Split. During the third year of the medical curriculum, students are exposed to numerical expressions embedded in a medical content (e.g. pathophysiology and pharmacology courses) and also attend a mandatory course in evidence-based medicine (addressing concepts such as number needed to treat, risk reduction, odds ratio, meta-analysis), which may have contributed to the high numeracy levels observed at their entrance to the fourth study year. Previous research also indicated that clinical maturity is an important factor which may contribute to the improvement of critical reflection and numerical understanding in health context, which may also be a reason why students at clinical years had higher scores [22]. Moreover, at the clinical part of the medical curriculum, it is to be expected that students perform calculations in a clinical context daily, and thus keep their high numeracy levels. It has been shown that participants who learn numeracy in a relevant context have greater chance of improvement of both objective and subjective numeracy [23]. This is supported by the evidence that professionals who perform everyday calculations in a clinical context have higher numeracy levels compared to medical students [24]. These findings emphasize the importance of implementation of clinical context in all numeracy- and statistics-related courses and programs in medical education.

## Conclusions

In conclusion, our study showed that both subjective and objective numeracy levels were higher among students at higher years of medical school. Research methodology and statistics course intervention did not make a difference between intervention and non-intervention group in objective numeracy scores, but the intervention group had higher subjective numeracy levels after 3 months. Although objective numeracy levels in medical student population are relatively high, courses in research methodology and biostatistics may possibly help to increase preferences towards numerical compared to verbal type of information and increase confidence in the use of numerical information in a medical context.

## Data Availability

The datasets used and analysed during the current study are available from the corresponding author on request.

## Abbreviations

NUMi: Numeracy Understanding in Medicine Instrument
SNS: Subjective Numeracy Scale
